# Development of a TaqMan qPCR Method for Detecting *Angiostrongylus cantonensis* (Rhabditida: Angiostrongylidae) Infection in Snails from Hainan Province, China

**DOI:** 10.3390/tropicalmed11020034

**Published:** 2026-01-23

**Authors:** Kun Wang, Tian Tian, Yunhai Guo, Muxin Chen, Xiaonen Wu, Zhiying Hou, Binbin Xie, Fanna Wei, Zhiheng Qi, Zhisheng Dang, Dingwei Sun, Yang Hong, Jun-Hu Chen, Yue Wang

**Affiliations:** 1 School of Public Health, Hangzhou Medical College, Hangzhou 310013, China; 2 National Key Laboratory of Intelligent Tracking and Forecasting for Infectious Diseases, National Institute of Parasitic Diseases, Chinese Center for Disease Control and Prevention (Chinese Center for Tropical Diseases Research), Shanghai 200025, China; 3 National Health Commission of the People’s Republic of China (NHC) Key Laboratory of Parasite and Vector Biology, Shanghai 200025, China; 4 World Health Organization (WHO) Collaborating Center for Tropical Diseases, Shanghai 200025, China; 5 National Center for International Research on Tropical Diseases, Shanghai 200025, China; 6 Hainan Tropical Diseases Research Center, Haikou 571199, China; 7 School of Basic Medical Sciences and Forensic Medicine, Hangzhou Medical College, Hangzhou 310013, China; 8 Hainan Provincial Center for Disease Control and Prevention, Haikou 571199, China

**Keywords:** *Angiostrongylus cantonensis*, snails, real-time polymerase chain reaction, diagnostic techniques, parasitological

## Abstract

*Angiostrongylus cantonensis* (*A. cantonensis*) is the primary causative agent of human angiostrongyliasis and is widely distributed in Southeast Asia and China, with increasing reports from the Americas. *Achatina fulica* (*A. fulica*), *Pomacea canaliculata* (*P. canaliculata*), and slugs constitute established intermediate hosts of *A. cantonensis*, whereas *Camaena hainanensis* (*C. hainanensis*) has been newly reported as a host species in Hainan. A TaqMan quantitative PCR (qPCR) method assay targeting a novel genomic region of *A. cantonensis* was developed to detect infection in 150 snails collected from Hainan Province, China. The assay was employed to detect the parasite larvae across various snail tissues (lung sac, mucus, and foot), and its performance was compared with conventional lung sac microscopy. Out of the 120 *A. fulica* examined, 75 tested positive using the qPCR assay, yielding a significantly higher detection rate than lung-sac examination (*p* < 0.05). Significant differences were also observed in the positivity rates across the three snail tissues (lung sac, mucus, and foot) (*p* < 0.05), with the lung sac showing the highest rate of infection. Importantly, the detection of *A. cantonensis* DNA in snail mucus highlights its potential for development as a non-invasive diagnostic sample. Additionally, *C. hainanensis* was identified as a new host of *A. cantonensis* in Hainan, suggesting its possible contribution to parasite transmission. The newly developed qPCR assay demonstrated superior sensitivity (reflected by lower Ct values) compared with previously published TaqMan qPCR methods. The established qPCR method provides a sensitive and non-invasive tool for detecting *A. cantonensis* in snails, and can be applied for monitoring and early warning of parasite prevalence and transmission.

## 1. Background

The World Health Organization (WHO) has classified *angiostrongyliasis* as an emerging infectious disease with significant global health implications [1,2]. *Angiostrongylus cantonensis* (Chen, 1935) (Rhabditida: Angiostrongylidae), hereafter *A. cantonensis*, is the primary causative agent of *angiostrongyliasis* and has been historically endemic in Southeast Asia, the Pacific Islands, and China. However, the parasite is now reported in other regions, including the Americas and Europe, where it is recognized as an important emerging public health concern. Most cases of eosinophilic meningitis (EOM) reported from endemic areas in Southeast Asia and the Pacific Islands are caused by *A. cantonensis* infection. Neuroangiostrongyliasis is one of the most common parasitic causes of EOM [3]. The disease may lead to headache, nausea, and vomiting due to elevated intracranial pressure. It can also result in neurological symptoms, including migratory pain, numbness, itching, and, in severe cases, coma or death [3]. A very high burden of larvae in humans often leads to more severe infection, increased mortality, permanent neurological complications, and long-term disability [4,5].

Adult *A. cantonensis* inhabit the pulmonary arteries of rats, their definitive host, where the female lays their eggs [6]. First-stage larvae migrate through the pulmonary capillaries to the alveoli, up through the respiratory tract to the pharynx, and are eventually expelled through the digestive tract with feces. Feces containing first-stage larvae are ingested by intermediate hosts such as *A. cantonensis*, where the larvae develop to the third stage. When rats consume these infected intermediate hosts, the third-stage larvae migrate to the brain and transform into fourth-stage larvae (L4). The L4 larvae subsequently move back into the venous system and ultimately reach the pulmonary arteries, where they mature into adult worms [7].

The intermediate hosts of *A. cantonensis* are snails and slugs, mainly including *Achatina fulica* (Bowdich, 1822), hereafter *A. fulica*, *Pomacea canaliculata* (Lamarck, 1819), hereafter *P. canaliculata*, and various species of slugs [8,9]. Humans are accidental hosts and are infected as a result of ingestion of raw or undercooked slugs or snails, ingestion of raw or undercooked paratenic hosts, or green leafy vegetables or fruits contaminated by intermediate hosts [10]. The first case of *A. cantonensis* infection in China was reported in Taiwan, China, in 1945, with subsequent outbreaks occurring in Zhejiang, Fujian, Guangdong, Beijing, and Yunnan [11,12,13].

A study of meta-analysis in China showed that the *A. cantonensis* infection rate of snail intermediate hosts was 6.89% (95% CI, 5.02–9.03%). Subgroup analysis showed that the infection rate in *A. fulica* was 18.76% (95% CI, 13.97–24.06%), the highest among the intermediate hosts. The infection rate of *A. fulica* in Hainan province was 13.15% (95% CI, 11.66–14.80%), the highest among all provinces included in the study [14]. The burden and infection rate of *A. cantonensis* were high in southeastern China, with Hainan Province recording the highest IgG-specific antibody positivity rate among all surveyed provinces [15]. Hainan Province has a warm and humid climate that supports diverse gastropod species, including *P. canaliculata*, *A. fulica*, and *Camaena hainanensis* (H. Adams, 1870), hereafter *C. hainanensis.* An investigation into the natural infection of *A. cantonensis* intermediate hosts in epidemic areas of Hainan is needed.

Monitoring and detection of *A. cantonensis* in snails are important for epidemiological surveys. Conventional parasitological detection techniques are ‘gold standard’ for the diagnosis of *A. cantonensis*, which include lung-microscopy, tissue homogenization, and enzymatic digestion methods. These methods are sensitive and cost-effective for laboratory parasite detection; however, they are time-consuming, inconvenient, and prone to false negatives. Molecular biology methods such as PCR, qPCR, Loop-mediated Isothermal Amplification (LAMP), and Recombinase Polymerase Amplification (RPA) are more sensitive [16,17,18,19,20], and methods like AcanR3990 qPCR have been established [21]. These methods are highly sensitive and suitable for epidemiological screening in endemic areas. In this study, a TaqMan qPCR method was developed, and it was used for the detection and identification of *A. cantonensis* in different tissues of intermediate hosts, including mucus.

## 2. Methods

### 2.1. Gastropod Collection, Maintenance, and Tissue Acquisition

Snail samples were collected from various cities in Hainan (n = 150), including 120 *A. fulica*, 20 *P. canaliculata*, and 10 *C. hainanensis* (Supplementary Figures S1–S3). Samples were obtained from Haikou, Qiongzhong, Baisha, and Sanya (Supplementary Figure S4). In Haikou, 90 samples were collected across three locations: Liufang Road (20 *A. fulica* and 10 *C. hainanensis*), Fengxiang Wetland Park (20 *A. fulica* and 20 *P. canaliculata*), and Renmin Park (20 *A. fulica*). In addition, 60 *A. fulica* samples were collected from Qiongzhong, Baisha, and Sanya, with 20 samples obtained from each city. All gastropods were maintained in the laboratory at room temperature. They were placed in plastic boxes containing soil and fed with lettuce once daily until use.

Prior to snail's dissection, a cotton swab was inserted through the aperture of the snail and manipulated repeatedly to collect mucus secretions. The swab head was snapped off and preserved in a 1.5 mL Eppendorf tube, which was stored at −20 °C for further genomic DNA extraction.

Gastropods were put in an ice bath (4 °C for 20–30 min) in order to induce relaxation and minimize pain. The shells were carefully cracked and then removed. The dead snails were washed thoroughly to get rid of the mucus and shell fragments on their body. The pulmonary sac occupies half of the mantle cavity, and the mantle was incised using scissors [22]. The lung sac was then excised and laid flat on a glass slide. All samples were dissected in the laboratory utilizing the lung microscopy method to determine their infection status.

A small muscle tissue sample, approximately 1 cm in length and 0.3 cm in width, was excised from the foot of the snails using scissors and preserved in a separate 1.5 mL Eppendorf tube, and then immersed in anhydrous ethanol. These samples were stored at −20 °C until use.

### 2.2. Lung Sac Checking by Microscopy

The entire structure of the lung sac was observed under a dissecting microscope. If larval nodules are present in the cyst wall tissue, they are carefully isolated and transferred onto a glass slide. A coverslip is then placed over the nodules, and gentle pressure is applied to squash them. The worm species were subsequently identified under the microscope [23,24]. L3 are larger at 425–550 µm in length, featuring a notched tail with a pointed terminal projection and often encased in sheathed exuviae from previous molts. Given the presence of nodules and the observation of larvae under the microscope, which matched the morphological characteristics of stage III *A. cantonensis* larvae, the samples can be classified as positive. Morphological examination was used as a preliminary screening method based on established descriptions, while final species identification relied on molecular confirmation.

### 2.3. DNA Extraction

The tissues were homogenized in 200 μL of Buffer GA (TIANamp Genomic DNA Kit, Beijing, China) on ice, after which 20 μL of Proteinase K was added and mixed thoroughly. Lysis was carried out at 56 °C for 1–3 h, with the samples gently inverted 2–3 times every hour. Following lysis, 200 μL of Buffer GB was added, mixed thoroughly, and the mixture was incubated at 70 °C for 10 min to obtain a uniform solution, then briefly centrifuged to remove droplets from the lid. Subsequently, 200 μL of ethanol (96–100%) was added and mixed thoroughly for 15 s. The entire mixture was then transferred to a CB3 Spin Column, centrifuged at 12,000 rpm for 30 s, after which the flow-through was discarded, and the spin column was placed into a clean collection tube. The remaining steps of the extraction procedure were carried out following the manufacturer’s protocol. DNA was eluted from the columns twice using 100 µL of TE buffer, yielding a final volume of 100 µL of DNA solution.

### 2.4. The qPCR Assay and Criteria for Determination

The target sequence was screened by bioinformatics analysis and verified through PCR detection experiments. Then, the TaqMan qPCR detection method targeting ITS1 was designed and established. The DNA amplification was conducted using the primers (Table 1). The qPCR assay was conducted in a final reaction volume of 20 μL, comprising 10 μL of 2× AceQ U+ Probe Master Mix (Vazyme, Nanjing, China), 0.4 μL each of AC-2N forward and reverse primers, 0.2 μL of TaqMan probe, 8 μL of nuclease-free water, and 1 μL of template DNA. The thermocycler was set to perform contaminant digestion at 37 °C for 2 min (one cycle), followed by pre-denaturation at 95 °C for 5 min (one cycle). This was succeeded by denaturation at 95 °C for 10 s and extension at 60 °C for 30 s, repeated for 40 cycles.

### 2.5. Criteria for Determination

Samples with a Ct value less than 37 were considered positive, those with a Ct value between 37 and 38 were classified as presumptive positive, and samples with a Ct value more than 38 were considered negative. Presumptive positive samples were retested, and if the Ct value was less than 38, they were classified as positive. If the Ct value remained between 37 and 38 or exceeded 38, the sample was classified as negative.

### 2.6. Comparison of the Detection Effect Between Lung Sac Detection and qPCR Assay

All the samples were examined by lung sac detection to determine the infection status. Genomic DNA was then extracted from the samples and analyzed using qPCR for molecular detection. The positive rates obtained from both methods were compared to evaluate their sensitivity and accuracy.

### 2.7. Comparison of the Detection Effect Between Two qPCR Assays

Previous TaqMan assay targeting ITS1 was used as a comparison. The previous qPCR assay was performed in a 20 μL reaction volume, containing 0.2 μM of each primer: AcanITS1F1 (5**′**-TTCATGGATGGCGAACTGATAG-3**′**) and AcanITS1R1 (5**′**-GCGCCCATTGAAACATTATACTT-3**′**), and 0.05 μM of the TaqMan probe AcanITS1P1 (5**′**-6-carboxyfluorescein-ATCGCATATCTACTATACGCATGTGACACCTG-BHQ-3**′**). Standard TaqMan cycling conditions were used, comprising 40 cycles of denaturation at 95 °C for 15 s and annealing/extension at 60 °C for 1 min [19]. Genomic DNA extracted from 10 positive and 10 negative samples detected through lung microscopy was selected for qPCR comparison.

### 2.8. Data Analysis

R Studio 4.4.1 was used for statistical analysis in this study. The main indicators assessed in the analysis included the positive rate and the negative rate. A positive rate represents the proportion of unknown infected samples that tested positive in an assay. Positive rate = The number of infected samples/Total number of samples tested. Negative rate represents the proportion of unknown infected samples that tested negative in an assay.

## 3. Results

### 3.1. Lung Sac Checking by Microscopy and Comparison Between qPCR

**Figure 1:**
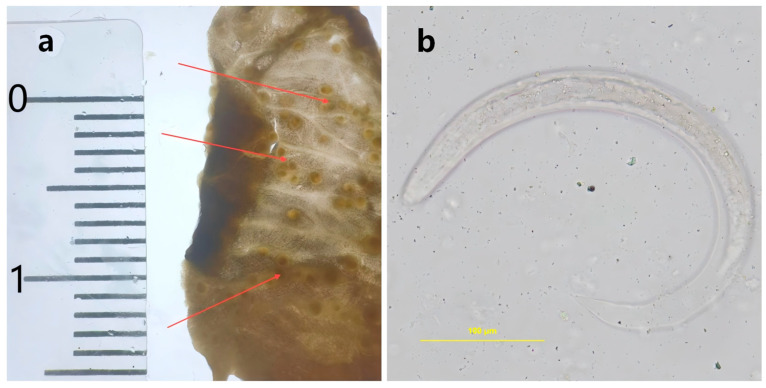
The nodules in the lung sac of *Achatina fulica*. (**a**): Larval nodules observed in the lung sac of *Achatina fulica*. (**b**): The third-stage larvae (L3) of *Angiostrongylus cantonensis* in the larval nodules.

Microscopic examination and qPCR showed different detection outcomes for *A. fulica* collected in Hainan. Among the examined 60 snails, 40 snails were identified as positive through microscopic examination (Figure 1). The qPCR confirmed positive results in all 40 samples. Additionally, eight positive cases were identified among the remaining 20 snails that were initially classified as negative (Table 2). The positive rate for microscopic examination was 66.7% (40/60, 95% CI, 53.31–78.31%), while the positive rate for qPCR was 80.0% (48/60, 95% CI, 67.67–89.22%). A statistically significant difference was observed between the two methods(χ^2^ = 30, *p* = 4.320463 × 10^−8^, *p* < 0.05). This result confirmed that qPCR has a higher sensitivity compared to lung sac microscopy. Therefore, molecular testing was adopted for all subsequent sample analyses.

### 3.2. Sampling and Validation

High infection rates of *A. cantonensis* were observed in *A. fulica* from multiple sampling sites in Hainan, including streets, public parks, and wetland parks. In Liufang road, 20 *A. fulica* were detected, and 15 tested positive, yielding a positive rate of 75.0% (15/20, 95% CI, 50.90–91.34%). At Park, 20 *A. fulica* were detected, and 11 tested positive, resulting in a positive rate of 55.0% (11/20, 95% CI, 31.52–76.94%). Furthermore, in a wetland that provides a more suitable habitat for *A. fulica*, 20 *A. fulica* were sampled, of which 16 were infected, yielding a positive rate of 80.0% (16/20, 95% CI, 56.33–94.27%).

None of the 20 *P. canaliculata* specimens collected tested positive. Ten *C. hainanensis* were collected, and 3 of these tested positive, resulting in a positive rate of 30.0% (3/10, 95% CI, 6.67–65.25%).

More samples were collected in other cities of Hainan, including Qiongzhong, Baisha, and Sanya (Table 3). Among these samples, Qiongzhong City exhibited a positivity rate of 65.0% (13/20, 95% CI, 40.78–84.61%), Baisha City 60.0% (12/20, 95% CI, 36.05–80.88%), and Sanya City 40.0% (8/20, 95% CI, 19.12–63.95%), indicating the high infection rate of *A. cantonensis* in intermediate hosts widely distributed throughout Hainan.

### 3.3. Comparison Between Different Tissues

Significant differences in positivity rates were observed among different tissues of snail samples (Table 4). The lung sac was recognized as the region with the highest larval concentration, yielding 75 positive results from 120 samples, and resulting in a positive rate of 62.5% (75/120, 95% CI, 53.20–71.17%). Given the close interaction between snail mucus and the human environment, we analyzed 120 mucus samples, yielding 54 positive results, and resulting in a positivity rate of 45.0% (54/120, 95% CI, 35.91–54.35%). Additionally, considering the local habit of consuming *A. fulica*, the foot tissue was examined, identifying 56 positive cases out of 120 samples, yielding a positivity rate of 46.7% (56/120, 95% CI, 37.51–55.99%).

The differences in positivity rates among the lung sac, muscle, and mucus groups were statistically significant (Chi-square test, χ^2^ = 8.9625, *df* = 2, *p* < 0.05). There is no significant difference in the positive rate between the mucus group and the foot tissue group (Chi-square test, χ^2^ = 0.016783, *df* = 1, *p* > 0.05). In contrast, the differences in the positive rates between lung sac and mucus (Chi-square test, χ^2^ = 6.7044, *df* = 1, *p* < 0.05), as well as between lung sac and foot tissue (Chi-square test, χ^2^ = 5.4458, *df* = 1, *p* < 0.05), were both statistically significant.

### 3.4. Comparison Between qPCR Method

The cross-reactivity of this method was evaluated by testing the Genomic DNA of other common parasitic species and bacteria: *Schistosoma mansoni*, *Echinococcus multilocularis*, *Plasmodium falciparum*, *Ancylostoma caninum*, *Clonorchis sinensis*, *Schistosoma japonicum*, *Escherichia coli*, *Salmonella enterica*, and *Escherichia albertii* (unpublished). No amplification was observed for any non-target species.

To compare this method with the previous method, 10 positive and 10 negative nucleic acid samples detected through lung microscopy were selected for evaluation. The results indicate that both methods detected all 10 positive samples successfully, with a relative sensitivity of 100%. The conventional method yielded negative results for all 10 negative samples, whereas our method detected 5 positive results among them. Additionally, for all positive samples, our method demonstrated lower Ct values compared to the conventional method, with an approximate difference of 4–5 Ct values (Table 5).

## 4. Discussion

In this study, the results demonstrated that qPCR confirmed positivity in all samples previously identified as positive through microscopy, yielding a relative sensitivity of 100%. Microscopy is limited only to gastropod species that have a lung sac and is highly dependent on the experience of the operator; certain larval nodules in the pulmonary sacs can be difficult to detect, increasing the likelihood of missed diagnoses and leading to an underestimation of the infection rate in intermediate hosts. Larval stages of *Angiostrongylus* species exhibit substantial morphological similarities, which limit the reliability of morphology-based identification alone. Therefore, qPCR proves to be a superior method for accurately assessing the infection levels in intermediate hosts.

Hainan Province features a tropical monsoon climate with minimal annual temperature variation, abundant rainfall, and dense vegetation, providing an ideal environment for both the definitive and intermediate hosts of *A. cantonensis* [25]. Detection of intermediate hosts using this method revealed that *A. fulica* is widely distributed and exhibits a high infection rate. Among the 120 samples collected, the positivity rate reached 62.5%, indicating a high infection level. Notably, these infected snails were found in the urban center, where some residents have a habit of consuming *A. fulica*, posing a substantial health risk to the local population. Beyond its local public health implications, *A. fulica* is recognized as one of the most invasive terrestrial gastropods worldwide. It has been reported as a competent intermediate host of *A. cantonensis* in multiple endemic and emerging regions, including Southeast Asia, the Pacific Islands, Africa, and the Americas. Its strong adaptability to urban and peri-urban environments, combined with frequent human contact and dietary exposure, greatly facilitates the maintenance and spread of the parasite. The high infection rate in *A. fulica* in this study further supports its critical role in sustaining the transmission cycle of *A. cantonensis* in endemic areas. The results revealed the necessity of targeted surveillance and control of this invasive species.

For other common intermediate hosts, *P. canaliculata* is a significant intermediate host of *A. cantonensis*. As an invasive species, it faces almost no natural predators in China and can rapidly proliferate, posing considerable environmental harm. *P. canaliculata* has been widely reported in southern China, Southeast Asia, Hawaii, California, and other regions. Its rapid expansion has raised increasing concern regarding its role in parasite dissemination. The low infection rate of *P. canaliculata* samples collected in this study may be attributed to its aquatic habitat, which may reduce its exposure to *A. cantonensis* larvae, as well as the small sample size. Therefore, further sampling and testing of *P. canaliculata* are necessary.

Additionally, *C. hainanensis* has been under-researched in terms of its role as an intermediate host of *A. cantonensis*. In this study, we detected 3 positive samples among 10 collected specimens, confirming that *C. hainanensis* can serve as an intermediate host for *A. cantonensis* and suggesting that a certain level of infection exists in Hainan. This also demonstrates the broad susceptibility of *A. cantonensis* to intermediate hosts. There are many potential intermediate hosts yet to be identified, as there are few reports of laboratory or natural infections in local snail species from different regions. Snails serve as intermediate hosts for a variety of parasites, so it is essential for diagnostic methods to have high specificity to avoid misdiagnosis due to cross-reactivity with other parasites.

Several studies demonstrate that small numbers of *A. cantonensis* L3 larvae are released into the mucus trail of some gastropod species [26,27]. Therefore, mucus is considered a potential pathway for the transmission of *A. cantonensis* [28]. The positive rate detected through mucus samples reached 45.06%, confirming that mucus can be used to detect *A. cantonensis* infection in intermediate hosts. However, due to the relatively low concentration of larvae in mucus compared to other tissues, the sensitivity of this method is reduced. Dissecting the lung sac requires specialized expertise and is time-consuming; sampling mucus samples only provides a more convenient and non-invasive method. In contrast, pulmonary dissection and subsequent qPCR testing, which require more skill and experience, are better suited for more precise epidemiological studies.

The comparison of the two methods indicates that both methods demonstrated a high sensitivity, detecting all 10 positive samples with 100% relative sensitivity, which confirms their effectiveness in identifying true positive cases. However, a notable difference was observed in the detection of negative samples. While the conventional method returned negative results for all 10 negative samples, our method identified 5 as positive. This suggests that our method may be more sensitive, potentially detecting low-level infections or borderline cases that the conventional method missed.

Additionally, the consistently lower Ct values obtained with our method across all positive samples indicate a higher amplification efficiency, as Ct values are inversely related to the quantity of target DNA. The observed difference of approximately 4–5 Ct units suggests that our method may offer superior sensitivity in detecting target sequences at earlier amplification cycles. This improved sensitivity could be advantageous in early diagnosis, and due to the consistent performance in specificity testing, this method has proven to be a reliable approach for detecting *A. cantonensis*.

The results from cross-reactivity of this method demonstrate that the assay exhibits high specificity and does not cross-react with genomic DNA from other parasites or bacteria commonly found in similar environments.

## 5. Conclusions

This study developed a high-sensitivity TaqMan qPCR method with higher sensitivity compared to microscopic examination. Confirming the high transmission burden of *A. cantonensis* in Hainan province, which led to the identification of *C. hainanensis* as a new intermediate host. Through the examination of different tissues of intermediate hosts, the lung sac exhibited the highest positive rate and can be considered a reliable target for accurate detection. Moreover, the detection of *A. cantonensis* DNA in both mucus and muscle tissues indicates potential routes for parasite transmission. Mucus offers potential for a non-invasive detection method and efficient field surveillance. Detection of the parasite in consumable muscle tissue highlights a significant risk of foodborne transmission.

## Figures and Tables

**Table 1:** The primers used in the present study.

Prime Name	Prime Sequence 5′-3′
AC-2N sense	GCGTTGTTGGTGATTATG
AC-2N antisense	CATGCGTATAGTAGATATGC
AC-2N probe	FAM-ATGATACTATCAGTTCGCCATCCATGAA-BHQ1

**Table 2:** Molecular detection record of intermediate hosts in Haikou city, Hainan province.

Region	*A. fulica* (Bowdich, 1822)			*P. Canaliculata* (Lamarck, 1819)			*C. hainanensis* (H. Adams, 1870)		
	Samples Tested (pcs)	Positive Cases	Positivity Rate (%)	Samples Tested (pcs)	Positive Cases	Positivity Rate (%)	Samples Tested (pcs)	Positive Cases	Positivity Rate (%)
Liufang Road	20	15	75.00%				10	3	30%
Renming Park	20	11	55.00%						
Fengxiang Wetland Park	20	16	80.00%	20	0	0%			
Total	60	42	70.00%	20	0	0%	10	3	30%

**Table 3:** Molecular detection record of intermediate hosts in Hainan province.

Region	*Achatina fulica*		
	Samples Tested (pcs)	Positive Cases	Positivity Rate (%)
Qiongzhong county	20	13	65.00%
Baisha county	20	12	60.00%
Sanya city	20	8	40.00%
Total	60	33	55.00%

**Table 4:** Molecular detection record of various tissues of snails.

Tissues	Positive Cases	Negative Cases	Positivity Rate (%)
Lung sac	75	45	62.50%
Mucus	54	66	45.00%
Foot	56	64	46.67%

**Table 5:** Comparison between conventional method and this method.

Samples NO.	Lung Detection	MeanCt1	MeanCt2
3	P	30.58	25.17
5	P	32.55	27.32
8	P	32.805	28.22
10	P	32.725	28.06
11	P	32.265	27.655
12	P	32.595	27.745
14	P	34.815	30.56
20	P	34.94	30.64
22	P	34.235	29.49
33	P	27.71	22.3
16	N	NoCt	37.715
18	N	NoCt	37.62
19	N	NoCt	37.47
27	N	NoCt	39.41
28	N	NoCt	38.37
29	N	NoCt	40.32
30	N	NoCt	37.105
31	N	NoCt	36.225
36	N	NoCt	36.16
37	N	NoCt	43.55
Blank control		NoCt	NoCt
Negative control		NoCt	NoCt
Positive control		31.775	27.445

MeanCt1 is the mean Ct value obtained from the experiment using the previously established method; MeanCt2 is the mean Ct value obtained from this method.

## Data Availability

The original contributions presented in this study are included in the article/Supplementary Materials. Further inquiries can be directed to the corresponding authors.

## Supplementary Materials

The following supporting information can be downloaded at: https://www.mdpi.com/article/10.3390/tropicalmed11020034/s1. The supplementary figures include photographs of intermediate host snails of *Angiostrongylus cantonensis*, namely *Achatina fulica*, *Pomacea canaliculata*, and *Camaena hainanensis*, as well as a schematic map showing the sampling locations across Hainan province, China. Figure S1: *Achatina fulica*; Figure S2: *Pomacea canaliculata*; Figure S3: *Camaena hainanensis*; Figure S4: Sampling locations arcoss Hainan province, China..

## Abbreviations

*A. cantonensis*	*Angiostrongylus cantonensis*
*A. fulica*	*Achatina fulica*
*P. canaliculata*	*Pomacea canaliculata*
*C. hainanensis*	*Camaena hainanensis*
qPCR	Quantitative polymerase chain reaction
Ct	Threshold cycle
