# Supplementary figures and images for: Development of a TaqMan qPCR Method for Detecting Angiostrongylus cantonensis (Rhabditida: Angiostrongylidae) Infection in Snails from Hainan Province, China
Source: Trop Med Infect Dis. 2026 Jan 23;11(2):34. doi: 10.3390/tropicalmed11020034 (PMC12945181; doi:10.3390/tropicalmed11020034)

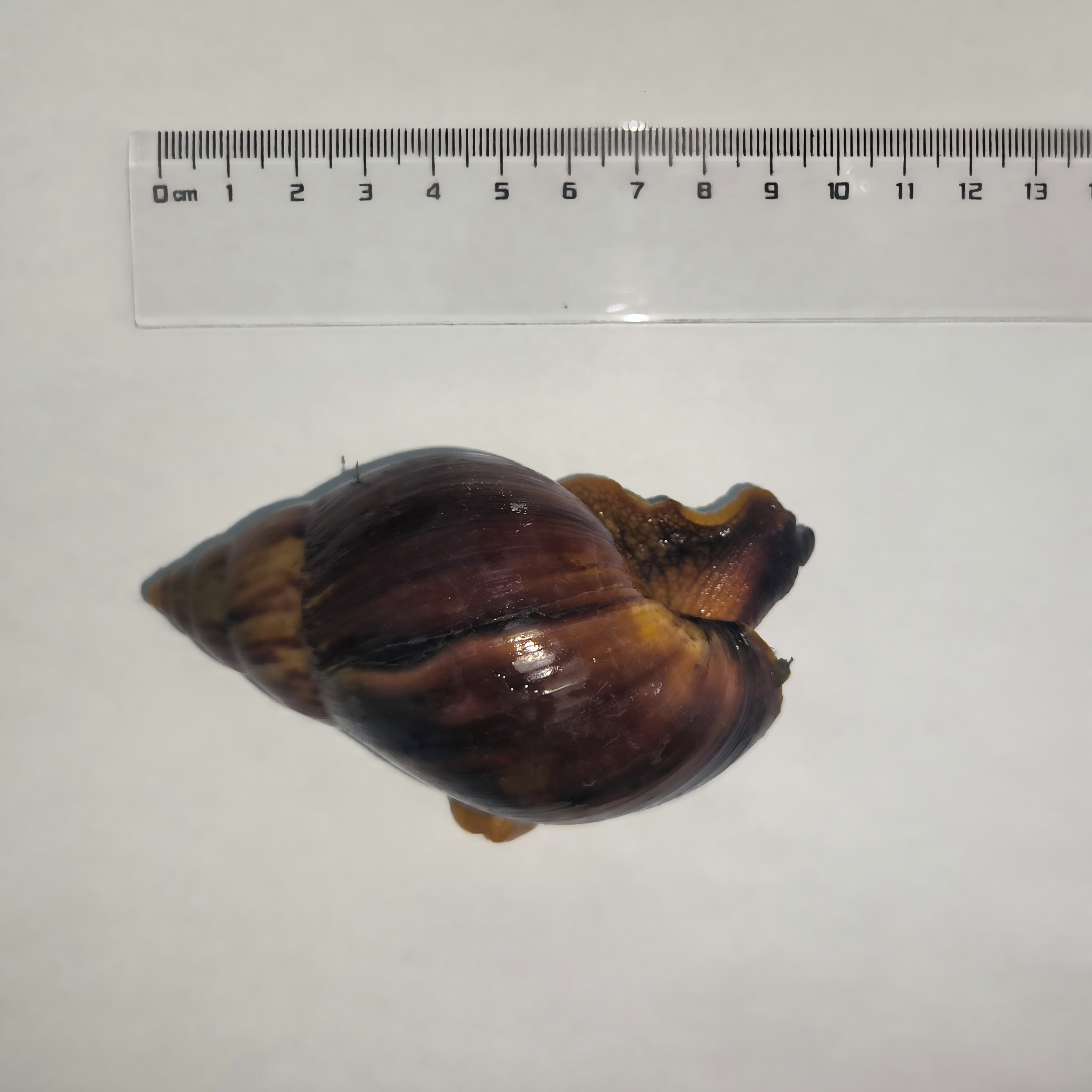

Supplement: Supplementary file 1 [file tropicalmed-11-00034-s001.zip › Supplementary Figure S1.png]

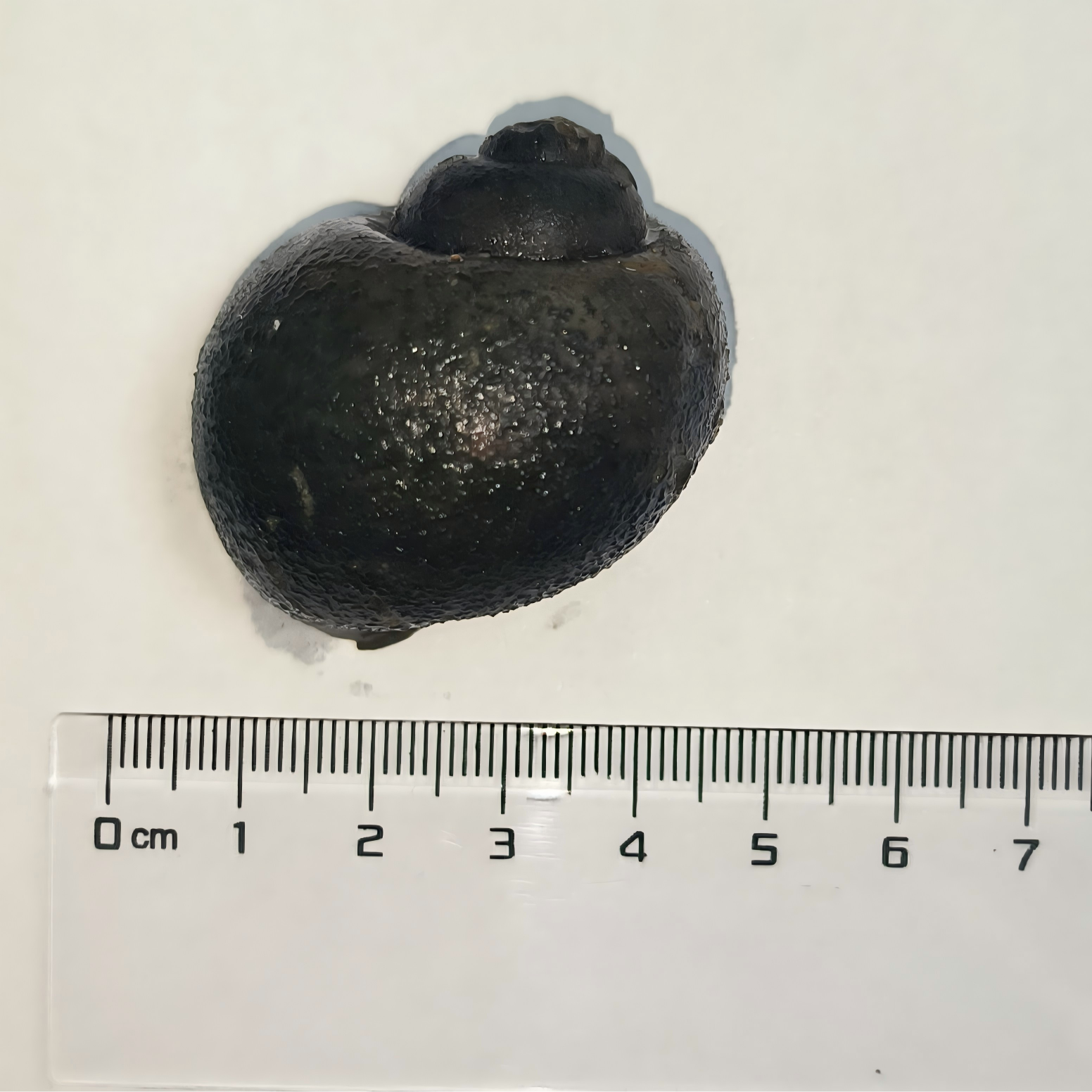

Supplement: Supplementary file 1 [file tropicalmed-11-00034-s001.zip › Supplementary Figure S2.png]

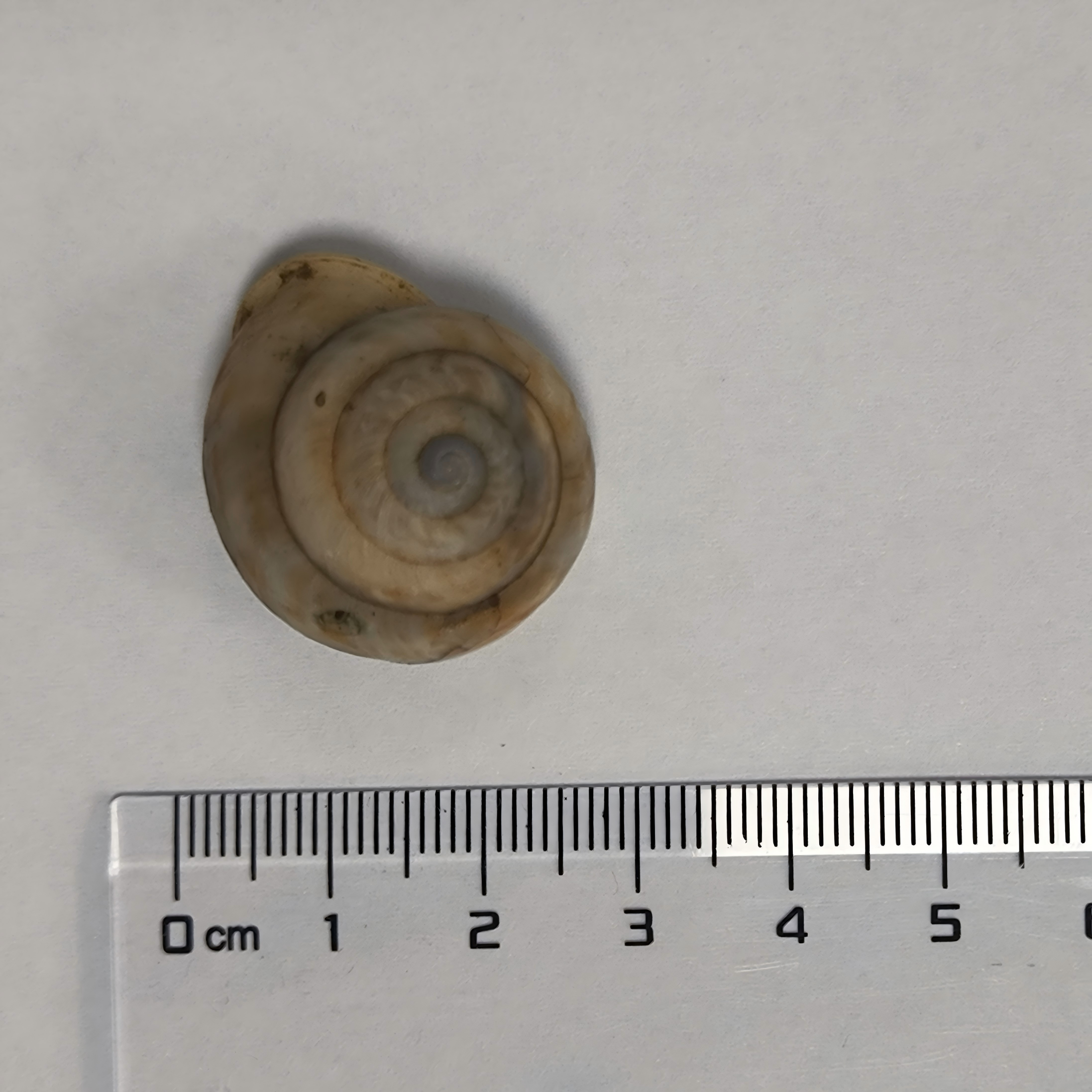

Supplement: Supplementary file 1 [file tropicalmed-11-00034-s001.zip › Supplementary Figure S3.png]

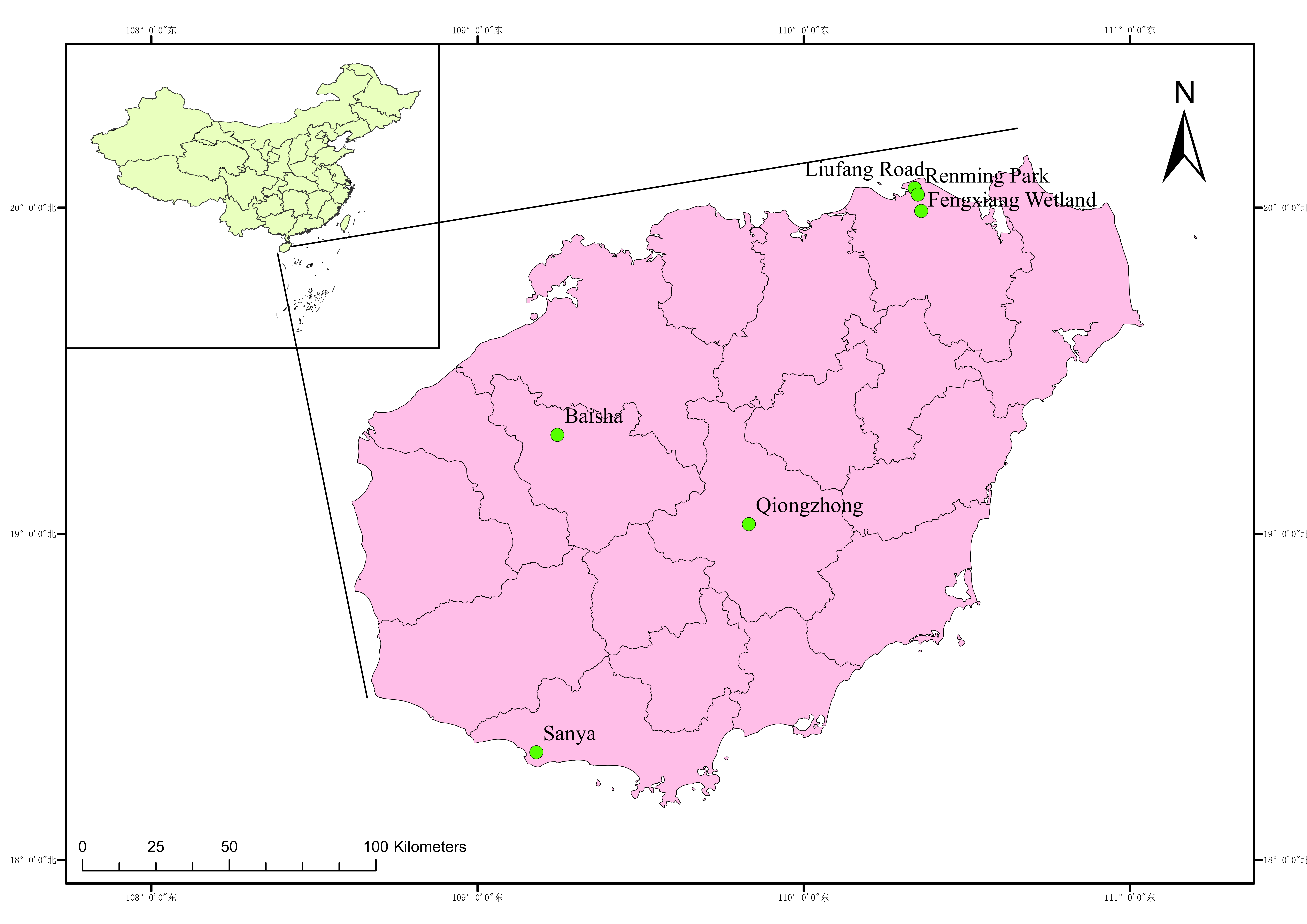

Supplement: Supplementary file 1 [file tropicalmed-11-00034-s001.zip › Supplementary Figure S4.png]
